# Effect of deep brain stimulation on brain network and white matter integrity in Parkinson's disease

**DOI:** 10.1111/cns.13741

**Published:** 2021-10-12

**Authors:** Li‐Chuan Huang, Li‐Guo Chen, Ping‐An Wu, Cheng‐Yoong Pang, Shinn‐Zong Lin, Sheng‐Tzung Tsai, Shin‐Yuan Chen

**Affiliations:** ^1^ Department of Medical Imaging Hualien Tzu Chi Hospital Buddhist Tzu Chi Medical Foundation Hualien Taiwan; ^2^ Department of Medical Imaging and Radiological Sciences Tzu Chi University of Science and Technology Hualien Taiwan; ^3^ Department of Medical Research Hualien Tzu Chi Hospital Buddhist Tzu Chi Medical Foundation Hualien Taiwan; ^4^ Cardiovascular and Metabolomics Research Center Hualien Tzu Chi Hospital Buddhist Tzu Chi Medical Foundation Hualien Taiwan; ^5^ Department of Neurosurgery Hualien Tzu Chi Hospital Buddhist Tzu Chi Medical Foundation Hualien Taiwan; ^6^ School of Medicine Tzu Chi University Hualien Taiwan

**Keywords:** deep brain stimulation, functional connectivity, graph theory, Parkinson disease, resting‐state functional MRI

## Abstract

**Aims:**

The effects of subthalamic nucleus (STN)‐deep brain stimulation (DBS) on brain topological metrics, functional connectivity (FC), and white matter integrity were studied in levodopa‐treated Parkinson’s disease (PD) patients before and after DBS.

**Methods:**

Clinical assessment, resting‐state functional MRI (rs‐fMRI), and diffusion tensor imaging (DTI) were performed pre‐ and post‐DBS in 15 PD patients, using a within‐subject design. The rs‐fMRI identified brain network topological metric and FC changes using graph‐theory‐ and seed‐based methods. White matter integrity was determined by DTI and tract‐based spatial statistics.

**Results:**

Unified Parkinson's Disease Rating Scale III (UPDRS‐ III) scores were significantly improved by 35.3% (*p* < 0.01) after DBS in PD patients, compared with pre‐DBS patients without medication. Post‐DBS PD patients showed a significant decrease in the graph‐theory‐based degree and cost in the middle temporal gyrus and temporo‐occipital part‐Right. Changes in FC were seen in four brain regions, and a decrease in white matter integrity was seen in the left anterior corona radiata. The topological metrics changes were correlated with Beck Depression Inventory II (BDI‐II) and the FC changes with UPDRS‐III scores.

**Conclusion:**

STN‐DBS modulated graph‐theoretical metrics, FC, and white matter integrity. Brain connectivity changes observed with multi‐modal imaging were also associated with postoperative clinical improvement. These findings suggest that the effects of STN‐DBS are caused by brain network alterations.

## Graphical abstract

This study uses multi‐modal images to explore the effectiveness of post‐deep brain stimulation (DBS) in Parkinson's disease (PD) patients. The result finds functional connectivity (FC) change in some brain areas. Moreover, Post‐DBS PD patients revealed a significant decrease in the graph‐theory‐based degree and cost in the middle temporal gyrus, temporo‐occipital part‐Right (toMTG‐R). Changes in topological metrics correlated with improved Beck Depression Inventory‐II and the FC changes with Unified Parkinson’s Disease Rating Scale‐III.

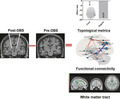

## 1 INTRODUCTION

Subthalamic nucleus (STN)‐deep brain stimulation (DBS) has been shown to improve both the motor symptoms and medication‐related complications of Parkinson's disease (PD).^1^, ^2^, ^3^, ^4^ A previous study found that the therapeutic synergy of DBS and levodopa was greater than the effect of either treatment alone.^5^ Recent studies further demonstrated that DBS is a therapeutic option even in the early stages of PD.^6^ However, DBS may exert adverse effects on non‐motor PD symptoms, such as impulsivity,^7^ depression,^8^ speech fluency, and cognition.^9^, ^10^ The mechanisms underlying the DBS‐induced amelioration of PD motor dysfunction and the effects of the combination of DBS with L‐DOPA on the neuronal network are elusive.

Resting‐state functional magnetic resonance imaging (rs‐fMRI) is widely used to study abnormal patterns of functional connectivity (FC) in PD patients. Functional changes in the cerebello‐thalamo‐cortical circuit are a hallmark of PD and have been associated with the major motor symptoms of the disease.^11^, ^12^ Decreased connectivity in the posterior putamen is the most consistent functional alteration associated with motor impairment,^13^ while the FC of the striatum with the cerebellum is reduced.^11^ Pathological FC also affects the cerebellothalamic and basal ganglia circuits in tremor‐dominant PD patients.^14^ Moreover, increased connectivity between the STN and the sensorimotor cortex is found in levodopa‐treated PD patients.^15^ Some studies that investigated the effect of DBS on brain FC demonstrated that it could increase functional brain connectivity within the cerebello‐thalamo‐cortical network^2^ and modulate effective connectivity within the cortico‐striato‐thalamo‐cortical loop.^16^ Another study showed that STN‐DBS ameliorates PD symptoms through normalization of the human functional connectome in PD.^17^ Most research focused on motor circuits in PD patients.^2^, ^16^ The brain areas mainly involved in these studies are the primary motor cortex (M1),^16^, ^18^, ^19^ supplementary motor area (SMA),^15^, ^19^ premotor cortex,^20^, ^21^ basal ganglia, thalamus, caudal SMA,^19^ cerebellum, and putamen.^22^ However, the effect of STN‐DBS on brain FC remains poorly understood, and little is known of its impact on the whole brain.

Graph theory is a powerful tool for characterizing the global topological organization of brain networks^23^, ^24^, ^25^ and for investigating abnormal functional brain networks in different stages of PD.^26^ A previous study found that PD patients exhibited lower global efficiency, higher clustering coefficients, and higher characteristic path lengths than healthy controls.^26^ Early‐stage drug‐naïve PD patients exhibited disruption of the whole‐brain topological organization (ie, decreased functional segregation and integration) of functional brain networks,^27^ which may contribute to preclinical changes in the cognitive process.^28^ At the node and connection level, PD patients exhibited reduced lengths of node centralities and connectivity, mainly not only in the temporal‐occipital regions, but also in the sensorimotor regions.^28^ No significant differences in intransitivity, characteristic path length, and degree were observed in the motor network related to the basal ganglia and cerebellum in PD patients, nor in the related network‐level values compared with normal controls.^29^ Levodopa modulates the global and local efficiency measures of small‐world topology in PD patients.^30^ However, the effect of STN‐DBS treatment on brain network graph theory in PD patients remains unclear. Diffusion tensor imaging (DTI) allows the measurement of fractional anisotropy and similarity. Previous studies on the use of DTI in PD have demonstrated abnormal fractional anisotropy in multiple white matter regions, particularly in the dopaminergic nuclei and pathways.^31^, ^32^ Hence, it has been used to assess both disease progression and the effects of treatment.^5^, ^33^ In the last few years, DTI has been applied for preoperative target localization in DBS surgery.^34^, ^35^ A recent study reported that the fibers connecting the electrode with the left prefrontal areas were associated with a worsening of depressive symptoms with STN‐DBS,^8^ suggesting that DTI could provide an additional means of assessing the evolution of psychiatric symptoms after surgery.^8^

Most rs‐fMRI research on STN‐DBS has focused on motor and non‐motor circuits in PD patients. The effects of STN‐DBS on brain FC and the topological metrics of graph theory in PD patients are unknown. Additionally, the impact of DBS on the fiber tract connectivity (integrity) profile of PD patients is unclear. We hypothesized that the benefit derived from STN‐DBS in PD relies on distributed brain networks and anatomical connections. Consequently, this study aimed to use multi‐modal imaging to compare the brain topological metrics, FC, and white matter integrity in levodopa‐treated PD patients’ pre‐ and post‐DBS.

## 2 MATERIALS AND METHODS

### 2.1 Subjects

From December 2016 to July 2019, 15 PD patients who had undergone STN‐DBS, had been followed‐up for at least 4 months, and showed significant motor improvement from the DBS were enrolled in this study (nine men and six women, mean age at DBS surgery: 58.2 ± 7.1 years). The inclusion criteria for STN‐DBS were as follows: a) PD patients who met the diagnostic criteria of the UK PD Brain Bank for the diagnosis of PD, b) at least 5 years disease duration, c) an L‐dopa challenge test showing an improvement of over 30% of the Unified Parkinson's Disease Rating Scale (UPDRS) motor score, and d) not demented and with mini‐mental state examination (MMSE) scores above the cutoff, in relation to educational status. Patients who did not meet these inclusion criteria were excluded from STN‐DBS. The pre‐DBS Unified Parkinson's Disease Rating Scale (UPDRS)‐III scores with medication “on” showed an improvement of 43.7% compared with the medication “off” state. The UPDRS‐III scores showed an improvement of 35.3% on the DBS effect. All patients received bilateral DBS electrodes (Model 3389, Medtronic), and pacemakers (implantable pulse generator, SC, Medtronic) were implanted. The stimulation parameters were set to produce optimal clinical responses. The average disease duration was 10.4 ± 3.9 years. All patients received bilateral STN‐DBS with a mean follow‐up time of 12 ± 3.8 months (range: 4‒16 months) in a within‐subject study design. The average medication dose of the patients, the levodopa equivalent daily dose (LEDD), was an average of 1141.3 mg/day (range: 520–2597.5) in pre‐DBS and 445.7 mg/day (range: 150–1130) in post‐DBS. A detailed description of the patient cohort and the stimulation parameters set to produce optimal clinical responses are shown in Table 1. The patients were not demented and had no major psychological problems. The accuracy of the surgery in STN target coordinates is shown in Table S1. Each participant provided written informed consent before the experiment. This study was approved by the Institutional Review Board of Tzu Chi General Hospital (Hualien, Taiwan; REC No: IRB 107–88‐B).

**TABLE 1:** Demographical details and stimulation parameters of the enrolled patients

Patient	Age onset	Disease duration (years)	Age surgery	Onset side	Amp (V)		PW (µs)		Rate (Hz)		Electrode		LEDD (mg/day)			MMSE Pre‐op	Follow‐up (months)
					CH1	CH2	CH1	CH2	CH1	CH2	CH1	CH2	Pre‐op	Post‐op	(%)		
1	52	12	64	R	3.4	3.2	60	60	130	130	0‐C+	0‐C+	765.0	300.0	60.8	23	7
2	57	6	63	R	2.8	2.5	60	60	130	130	1‐C+	1‐C+	520.0	210.0	59.6	27	10
3	37	20	57	L	2.9	3.6	60	60	130	130	1‐C+	1‐C+	1191.3	465.0	61.0	28	14
4	52	9	61	L	3.2	3.2	60	60	130	130	1‐C+	1‐C+	1326.3	150.0	88.7	27	16
5	59	7	66	Bil	3.7	3.4	60	60	130	130	1‐C+	1‐C+	1396.3	830.0	40.6	17	13
6	61	8	69	L	2.8	2.8	60	60	130	130	1‐C+	2‐C+	1695.0	1130.0	33.3	28	5
7	44	9	53	L	2.4	3.4	60	60	130	130	1‐C+	1‐C+	600.0	620.0	−3.3	27	12
8	38	9	47	L	3.3	3.5	60	60	130	130	2‐C+	1‐C+	1020.0	510.0	50.0	30	15
9	45	10	55	L	2.3	3.6	60	60	130	130	1‐C+	1‐C+	1370.0	382.5	72.1	30	15
10	41	10	51	L	3.7	3.9	60	60	130	130	2‐C+	1‐C+	1530.0	415.0	72.9	28	14
11	46	14	60	R	2.7	1.6	60	60	130	130	1‐C+	1‐C+	800.0	300.0	62.5	25	15
12	54	7	61	L	3.3	3.4	60	60	130	130	1‐C+	1‐C+	577.5	520.0	10.0	25	13
13	45	9	54	R	3.6	3.2	60	60	130	130	1‐C+	1‐C+	800.0	220.0	72.5	28	14
14	57	9	66	R	2.7	2.8	60	60	130	130	1‐C+	1‐C+	930.0	232.5	75.0	27	13
15	29	17	46	L	2.8	2.3	60	60	130	130	2AB‐C+	2AB‐C+	2597.5	400.0	84.6	29	4
Mean	47.8	10.4	58.2		3.0	3.1	60.0	60.0	130.0	130.0			1141.3	445.7	56.0	26.6	12.0
SD	9.2	3.9	7.1		0.5	0.6	0.0	0.0	0.0	0.0			545.3	259.8	26.2	3.2	3.8

Abbreviations: Amp, amplitude; Bil, bilateral; CH, channel; L, left side; MMSE, mini‐mental state examination; PW, pulse width; R, right side.

### 2.2 Clinical assessments

The UPDRS was used to evaluate the patient's mentation, behavior, and mood (Part I), activities of daily living (Part II), and severity of motor symptoms (Part III) before surgery (pre‐DBS) and at the time of follow‐up. Cognition and neuropsychological functions were also rated with the MMSE and Beck Depression Inventory (BDI‐II). At the post‐DBS follow‐up, the UPDRS was evaluated in four conditions: on and off medication, and with and without DBS stimulation. Pre‐DBS_MedOFF_ was defined by the worst off‐PD medication state, while Pre‐DBS_MedON_ was defined by the best on‐PD medication state. Post‐DBS_ON/MedON_ was defined by the best “on” state in which DBS was switched on and the patient was on medication, while Post‐DBS_ON/MedOff_ was defined by the state in which DBS was switched on, although the patient was off medication. The Hoehn & Yahr (H&Y) stage and L‐dopa equivalent daily dose (LEDD) were also recorded before and after DBS, for comparison.

### 2.3 Magnetic resonance imaging acquisition

#### 2.3.1 Resting‐state functional magnetic resonance imaging and Diffusion tensor imaging

Resting‐state functional magnetic resonance imaging, three‐dimensional (3D) T1‐weighted MRI, and DTI were performed before DBS surgery and at the time when patients had achieved stable postoperative status. At pre‐DBS, these images were obtained during the patient's best on‐medication status. At the time of follow‐up, patients were in an on‐medication state, and DBS was switched off before entering the MRI suite. All scans were performed in a 1.5‐T MRI scanner (Magnetom Aera; Siemens AG) using a 20‐channel phase‐array head coil. The rs‐fMRI was acquired by using an echo‐planar imaging (EPI) sequence with a repetition time (TR)/echo time (TE)/flip angle = 2980 ms/40 ms/90°, field‐of‐view (FOV) = 22 cm, matrix: 64 × 64, 36 axial slices covering the whole brain, voxel size 3.4 × 3.4 × 3.4 mm^3^, and 120 volumes, in a 6.08‐min scan. Patients were instructed not to focus their minds on specific thoughts and to keep awake with their eyes closed during the acquisition. Head motion was minimized by using foam pads. For registration purposes, 3D T1‐weighted anatomical images were acquired with a magnetization‐prepared rapid gradient echo method with the following parameters: TR/TE/inversion time/flip angle = 2200 ms/2.9 ms/900 ms/8°, matrix = 256 × 224, FOV = 25 cm, and 176 sagittal slices with 1‐mm thickness. DTI was acquired with a spin‐echo EPI sequence and the following parameters: TR/TE = 4000/97 ms, matrix = 128 × 128, FOV = 23 cm, 30 directions, b = 1000 s/mm^2^, and slice thickness = 5 mm.

#### 2.3.2 Resting‐state functional MRI image pre‐processing

All resting‐state fMRI datasets were processed using SPM12 (http://www.fil.ion.ucl.ac.uk/spm) and MATLAB 2018 (The MathWorks Inc.). Standard pre‐processing included realignment, slice‐timing correction, motion correction, normalization with respect to the EPI template (sampling size matched to the native 2‐mm isotropic resolution) provided by SPM, and 8‐mm Gaussian smoothing. The structural scan was normalized with respect to the T1 template of SPM. Finally, image segmentation was performed on the T1‐weighted images to yield gray matter, white matter, and cerebrospinal fluid (CSF) masks in normalized space. Time‐series data were high‐pass filtered (1/128 Hz), and 14 nuisance parameters (six movement parameters and their first derivative, white matter, and CSF) were regressed out. We performed quality checking procedures before analyzing images. When patients exhibited head motion of more than 1.5 mm in any of the x, y, or z directions, more than 1.5° of any angular dimensions were discarded.

#### 2.3.3 Resting‐state functional MRI data FC and graph‐theory analysis

The correlation of FC networks and topological metrics was analyzed by the CONN 18b connectivity toolbox^36^ (http://www.nitrc.org/projects/conn) running in MATLAB. The analysis of graph theory and topological metrics has been detailed previously.^25^ After band‐pass filtering (0.01 < f < 0.08 Hz), denoising was performed using aCompcor (anatomical component‐based noise correction method)^37^ to eliminate non‐neuronal contributions from the white matter and CSF. The denoising step also included the regression of time points flagged as outliers due to motion, along with the seven realignment (three translational, three rotational, and one composite motion) parameters and their first‐order derivatives. The global topological metrics of the network characteristics included the global efficiency, local efficiency, betweenness centrality, cost, average path length, clustering coefficient, and degree^36^ test on 164 seed regions (Table S2). Regions of interest (ROIs) were defined by the default atlas provided with the CONN, which included 164 parcellations (91 cortical and 15 subcortical parcellations from the Harvard‐Oxford Maximum Likelihood atlas, 26 cerebellar parcellations from the automated anatomical labeling atlas, and 32 ROIs of networks defined from CONN's independent component analyses of the HCP dataset). The correlation matrix was constructed of 164 × 164 ROIs. The entire matrix of ROI‐to‐ROI FC values was computed for each subject using a bivariate correlation measure, and a threshold at a fixed network‐level cost value (0.15)^38^ to define an undirected graph that characterized the entire network of functional connections between these ROIs. The pre‐DBS and post‐DBS connectivity differences in PD patients were estimated by specifying a condition contrast of [−1 1] in the second‐level analysis of the CONN.

#### 2.3.4 Diffusion tensor imaging data pre‐processing and TBSS analysis

For each subject, DTI images were converted from DICOM to NIFTI format with MRIcron (http://people.cas.sc.edu/rorden/mricron/index.html). The diffusion data were corrected for eddy currents and movement distortions using the Eddy Correct tool in FSL. Non‐brain parts were removed from all images using the Brain Extraction Tool. Diffusion tensors at each voxel were fitted by the algorithm included in the Diffusion Toolbox of the FMRIB Software Library (FSL v. 6.0, www.fmrib.ox.ac.uk/fsl). Fractional anisotropy (FA), mean diffusivity (MD), axial diffusivity (AD, λ1), and radial diffusivity (RD, [λ2 + λ3]/2) to the principal diffusion direction were computed for the whole brain.

The tract‐based spatial statistics (TBSS) tool^39^ was used to perform a voxel‐wise statistical analysis of the diffusion tensor maps. FA images of all subjects were aligned in the 1 × 1 × 1 mm^3^ standard Montreal Neurological Institute (MNI152) space via the FMRIB58_FA template using nonlinear registration. The mean FA image was created and thinned to create the mean FA skeleton, which represented the centers of all tracts common to the groups. This skeleton was threshold at FA > 0.20.^40^, ^41^ Each subject's aligned FA map was then projected onto this skeleton by assigning each point on the skeleton with a maximum FA in a plane perpendicular to the local skeleton structure. The resulting skeletons were analyzed using voxel‐wise statistics. Voxel‐wise cross‐subject statistics analysis was performed using 5000 permutations and tested with the threshold‐free cluster enhancement (TFCE) approach.^40^ Specific details of the analyses are given in a previous report.^39^

### 2.4 Statistical analysis

All data were tested to ensure they were normally distributed. If a group of data did not exhibit a normal distribution, a Student *t* test or one‐way ANOVA test was applied with a non‐parametric equivalent. We use repeated measure analysis of variance to test UPDRS‐III scores, including factors such as treatment method (pre vs. post), and post hoc Dunn's tests to identify significant differences from each treatment state using Prism 6 (https://www.graphpad.com). Additionally, H&Y stage, BDI‐II, MMSE, and LEDD scores in the same patient were assessed pre vs. post by paired *t* test. A paired *t* test was used to determine where graph theory and FC changes occurred between the pre‐DBS and post‐DBS in the same PD patient. We also assessed the connections and retained only the significant connections. The resulting statistical parametric maps were corrected for multiple comparisons using the false discovery rate (FDR) approach with *p* < 0.05 considered significant. We did a correlation analysis based on UPDRS‐III and BDI‐II improvement correlation to the dependent variable topological metrics and FC to identify the post‐DBS functional connectivity changes and topological metrics correlation to clinical improvement.

Whole‐brain FA and MD images were compared with pre‐DBS and post‐DBS differences. The FA and MD were compared between the pre‐and post‐DBS conditions with paired *t* tests. The significance threshold was set at *p* < 0.05, FDR‐corrected for multiple comparisons performed by permutation test with TFCE.

## 3 RESULTS

### 3.1 Effect of DBS on motor disability and non‐motor symptoms of cognition and language

**FIGURE 1:**
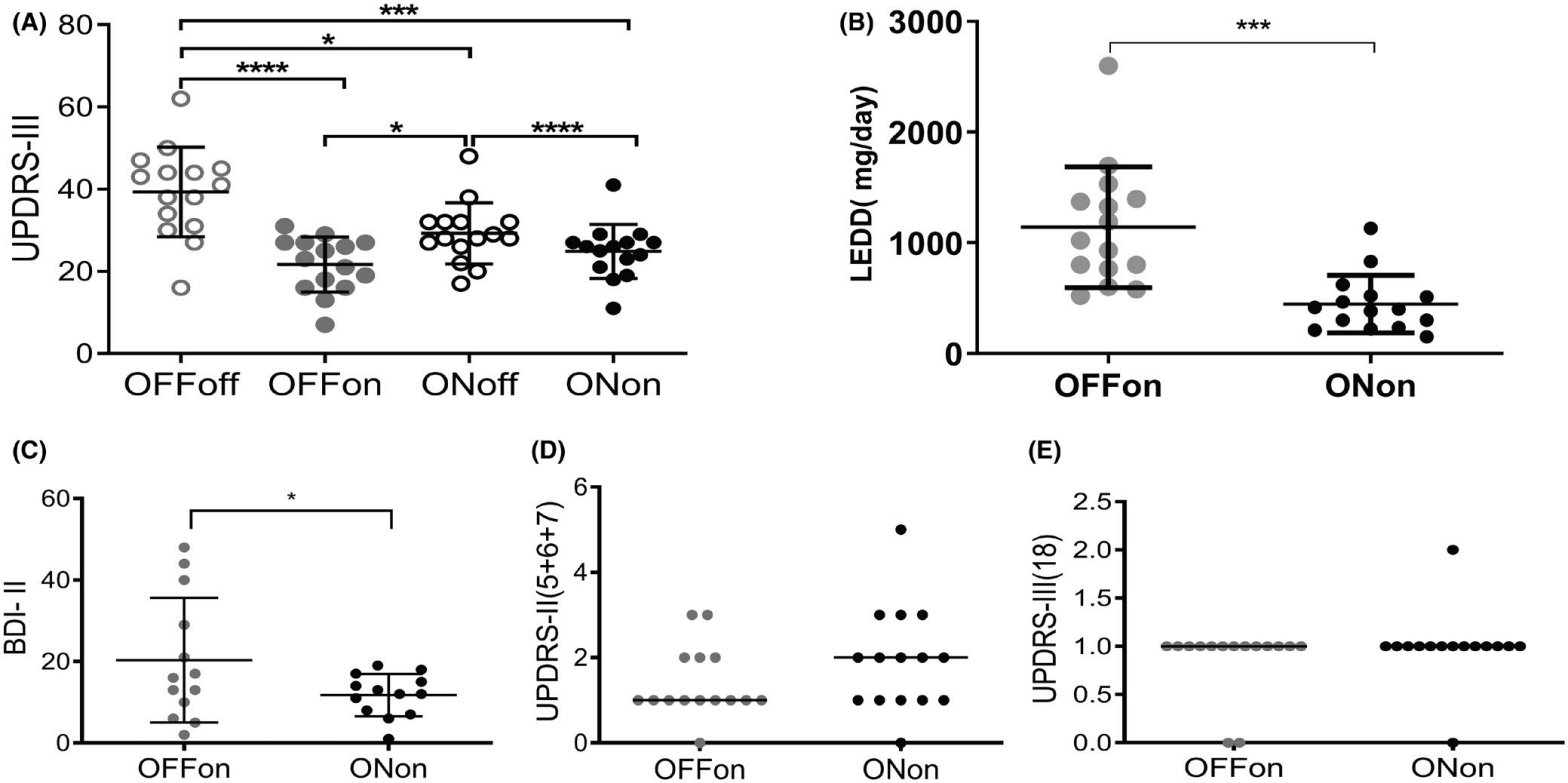
(A) Unified Parkinson Disease Rating Scale‐part III motor disability (best score = 0, worst score = 108) was evaluated in the pre‐DBS_MedOFF_ (OFFoff), pre‐DBS_MedON_ (OFFon), post‐DBS_ON_/_MedON_ (ONon), and post‐DBS_ON/MedOFF_ (ONoff) states. (B) L‐dopa equivalent daily dose (LEDD) in the pre‐DBS_MedON_ and post‐DBSON/_MedON_ states; (C) BDI‐II score, (D) UPDRS part II language (items 5 + 6 + 7) and (E) part III speech (item 18) in the pre‐DBS_MedON_ and post‐DBS_ON/MedON_ states. Abbreviations: UPDRS‐III, Unified Parkinson's Disease Rating Scale motor score‐part III; STN, subthalamic nucleus; DBS, deep brain stimulation; Pre‐DBS_MedOFF_., before electrode implantation for DBS and without medication (L‐dopa); pre‐DBS_MedON_, before electrode implantation for DBS and with medication; post‐DBS_ON/MedON_, after DBS treatment for at least 4 months and with medication. (*p < 0.05; ***p = 0.0001; ****p < 0.0001)

Deep brain stimulation on (DBS_ON_) alone improved motor disability significantly, by 35.3% (UPDRS part III, Figure 1A). The LEDD was significantly decreased by 56.0% in the post‐DBS compared with the pre‐DBS state (Figure 1B). In the medication‐on state, there was no significant difference between pre‐DBS and post‐DBS_ON_ in UPDRS‐III (21.7 ± 6.7% vs. 24.9 ± 6.6%, respectively). Non‐motor symptoms of depression were significantly improved after DBS (BDI‐II, pre‐DBS/post‐DBS: 19.9 ± 16.5/12.1 ± 6.5, *p* < 0.05; Figure 1C). However, cognition (CASI, pre‐DBS/post‐DBS: 87.4 ± 12.5/86.5 ± 14.1; MMSE, pre‐DBS/post‐DBS: 26.6 ± 3.2/26.5 ± 4.1) and language functions (UPDRS‐Ⅱ items 5+6+7 pre‐DBS/post‐DBS: 1.4 ± 0.8/1.9 ± 1.2, and UPDRS‐Ⅲ item 18 pre‐DBS/post‐DBS: 0.9 ± 0.4/1.0 ± 0.4; Figure 1D, E) were not significantly changed after DBS, although the language showed a trend of deterioration.

### 3.2 Graph theory at pre‐DBS and post‐DBS and correlation with clinical changes

**FIGURE 2:**
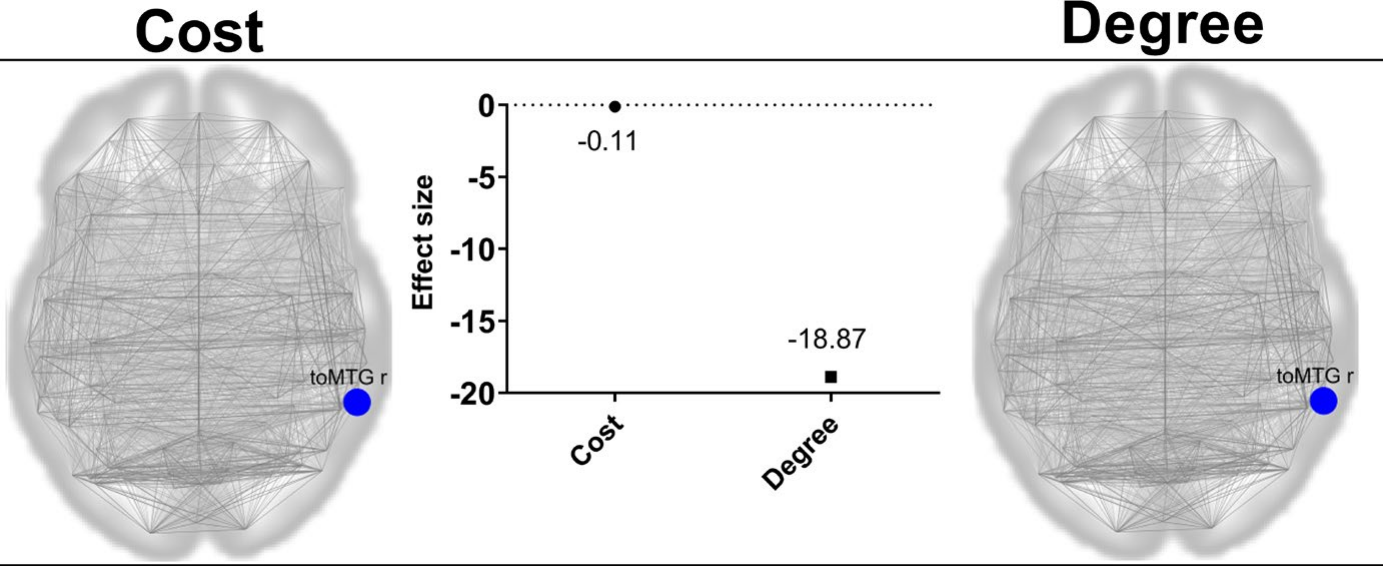
Network topological alterations observed in PD patients pre‐DBS vs post‐DBS (contrast: post‐DBS >pre‐DBS). Graph theory reveals post‐DBS treatment changes in network topological metrics in the middle temporal gyrus, temporo‐occipital part‐Right (toMTG‐R): x, y, and z = (58, −49, and 2); both cost and degree were decreased compared with pre‐DBS. Blue dot: region of interest with decreased topological metrics (*paired *t* test, *p*_FDR_ < 0.05)

**FIGURE 3:**
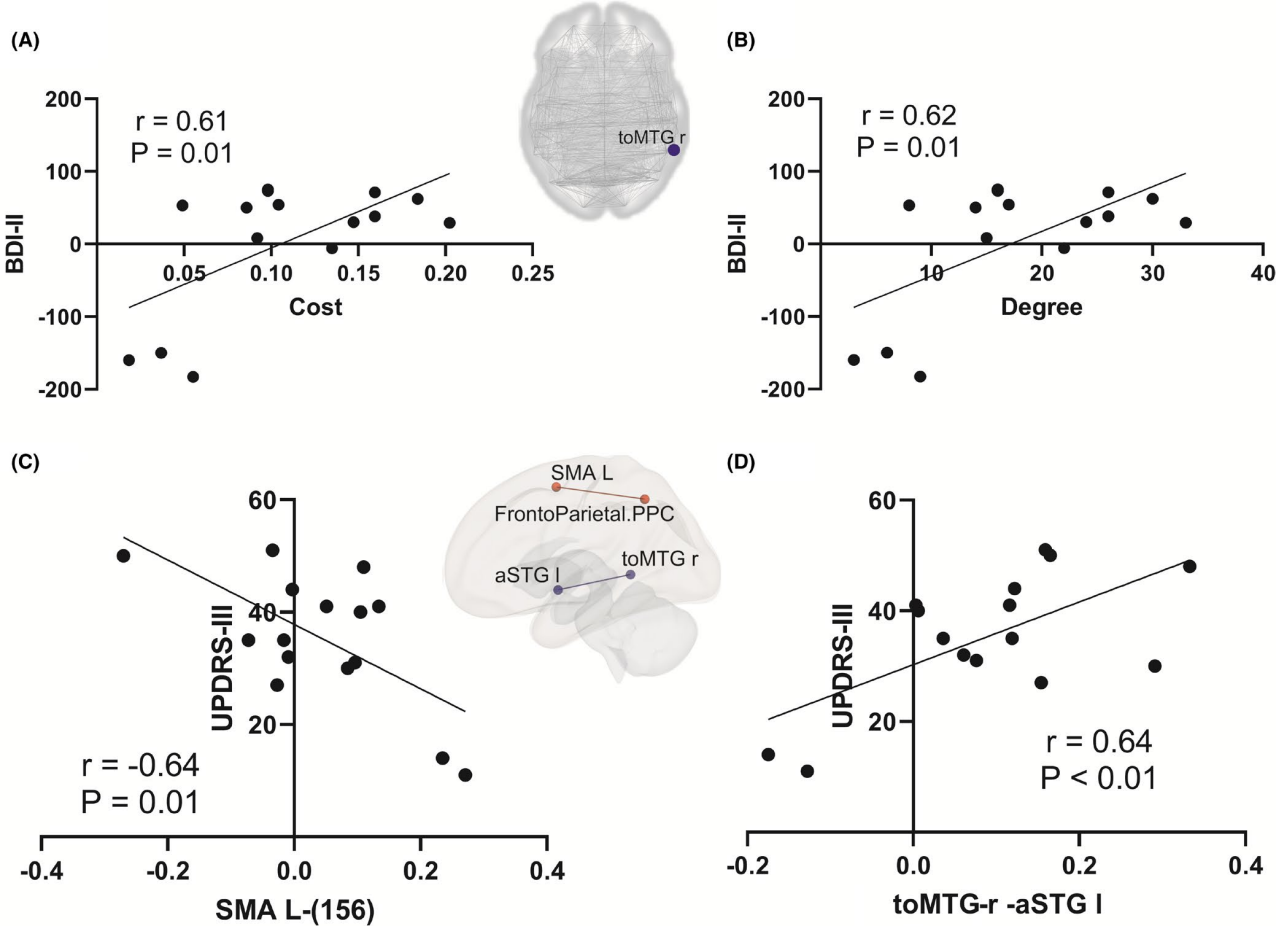
Topologic metrics and functional connectivity (FC) changes correlated with BDI‐II and UPDRS‐III score improvement in post‐DBS. The BDI‐II improvement was correlated with cost and degree decrease at toMTG‐R (A, B). The UPDRS‐III improvement showed a negative correlation with the FC increase in SMA L‐(156) (C) and a positive correlation with the FC decrease in toMTG‐R ‐aSTG L (D). * 156 = networks. FrontoParietal‐PPC L

In graph theory, the cost and degree were significantly decreased (cost: pre‐DBS/post‐DBS: 0.22 ± 0.08/0.11 ± 0.0; degree: pre‐DBS/post‐DBS 35.73 ± 13.34/17.67 ± 8.9) in the middle temporal gyrus and temporo‐occipital part‐Right (toMTG‐R) of patients post‐DBS compared with the pre‐DBS values. Conversely, global efficiency, local efficiency, betweenness centrality, and average path length did not differ between the two conditions (Figure 2). In post‐DBS, both cost and degree topological metrics in toMTG‐R were correlated with BDI‐II improvement (r = 0.62; *p* = 0.014*; Figure 3A, B).

### 3.3 Functional connectivity changes at post‐DBS and pre‐DBS and correlation with clinical changes

**FIGURE 4:**
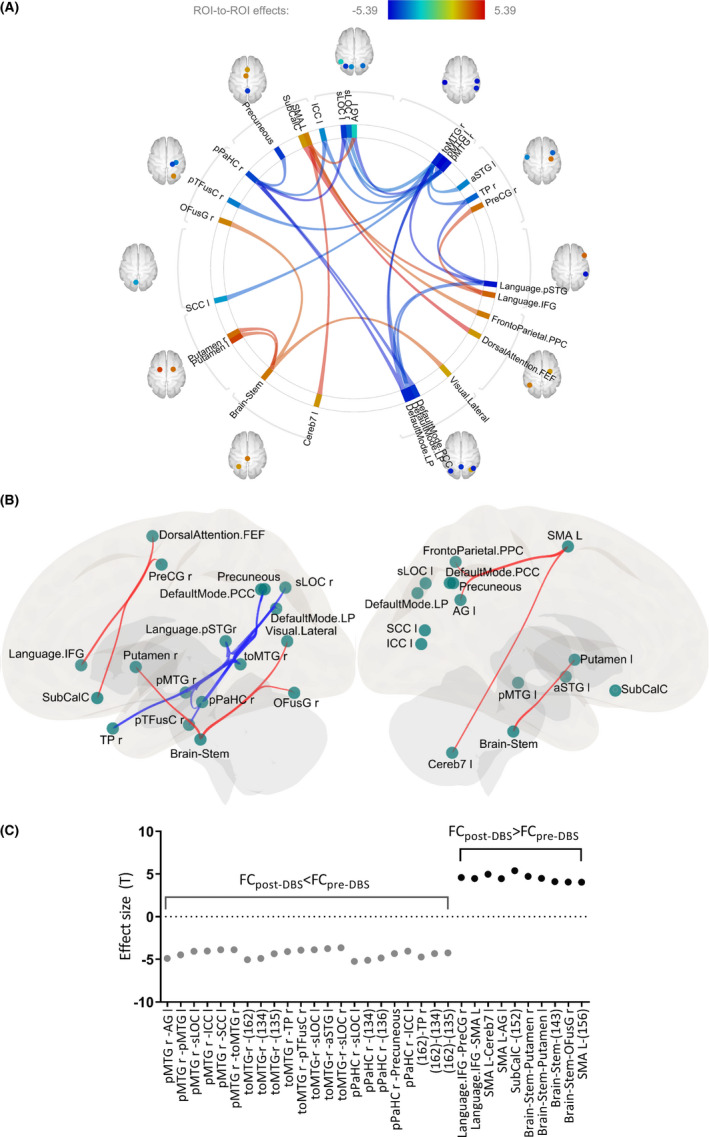
Functional brain changes in PD patients post‐DBS compared with pre‐DBS shown in the connectome ring (A). The changes between the regions of interest (ROIs)‐ROIs are shown in the 3D right and left hemispheres (B). Brain network FC decreased (white bars) and increased (black bars) between ROI‐ROI after STN‐DBS (C). *162: Networks Language pSTG‐R; 134: Networks Default Mode LP‐L; 135: Networks Default Mode LP‐R; 136: Networks Default Mode PCC; 152: Networks Dorsal Attention FEF‐R; 143: Networks Visual Lateral‐R; 156: Networks‐FrontoParietal PPC L. Red line: FC increase in post‐DBS patients compared with pre‐DBS. Blue line: FC decreased in post‐DBS patients compared with pre‐DBS

Figure 4 and Table 2 show four principal increases and four critical decreases in brain FC. Brain FC of the Language inferior frontal gyrus (IFG) with the PreCG‐R and SMA‐L; SMA‐L with Cereb7‐L and AG‐L; SubCalC with Networks Dorsal Attention FEF‐R; and Brainstem with Putamen‐R, Putamen‐L, Networks Visual Lateral‐R, and OFusG‐R was increased (red lines; Figure 4).

**TABLE 2:** Results of alteration in FC of brain regions are showing between‐group (post‐DBS treatment vs. pre‐DBS)

ROI‐ROI	FC	T value	*p*‐FDR
post‐DBS>pre‐DBS			
Networks. DorsalAttention.FEF R‐SubCalC	↑	5.39	0.0155
Brain‐Stem‐Networks. Visual. Lateral R		4.11	0.0481
Brain‐Stem‐OFusG R		4.06	0.0481
Brain‐Stem‐Putamen L		4.49	0.0413
Brain‐Stem‐Putamen R		4.72	0.0413
Language.IFG–PreCG‐R		4.59	0.0424
Language.IFG ‐SMA R		4.48	0.0424
SMA L‐ Networks. FrontoParietal‐PPC L		4.04	0.0499
SMA L‐AG L		4.45	0.0298
SMA L‐Cereb7‐L		4.97	0.0298
post‐DBS<pre‐DBS			
Networks. DefaultMode.LP L‐Networks. Language.pSTG R	↓	−4.32	0.0386
Networks. Language.pSTG R‐Networks. DefaultMode.LP R		−4.25	0.0331
Networks. Language.pSTG R‐TP R		−4.73	0.0265
pMTG R‐AG L		−4.89	0.0386
pMTG R‐ICC L		−4.02	0.0461
pMTG R‐pMTG L		−4.48	0.042
pMTG R‐SCC L		−3.87	0.0461
pMTG R‐sLOC L		−4.06	0.0461
pMTG R‐toMTG‐R		−3.87	0.0461
pPaHC R‐Networks. DefaultMode.LP L		−5.11	0.013
pPaHC R‐Networks. DefaultMode.PCC		−4.85	0.0139
pPaHC R‐ICC L		−4.04	0.0395
pPaHC R‐Precuneous		−4.33	0.0284
pPaHC R‐sLOC L		−5.25	0.013
toMTG‐R‐Networks. DefaultMode.LP L		−4.9	0.0192
toMTG‐R‐Networks. DefaultMode.LP R		−4.34	0.0369
toMTG‐R‐Networks. Language.pSTG R		−5.04	0.0192
toMTG‐R ‐aSTG L		−3.65	0.0497
toMTG‐R‐pTFusC R		−3.92	0.0461
toMTG‐R‐sLOC L		−3.74	0.0497
toMTG‐R‐sLOC R		−3.63	0.0497
toMTG‐R‐TP R		−4.09	0.0447

Abbreviations: ↑, increase; ↓, decrease; L, Left; R, right.

Conversely, the FC of the pMTG‐R with AG‐L, pMTG‐L, sLOC‐L, ICC‐L, SCC‐L, and toMTG‐R; toMTG‐R with Networks Language pSTG‐R, Networks Default Mode LP‐L, Networks Default Mode LP‐R, TP‐R, pTFusC‐R, sLOC‐L, aSTG‐L, and sLOC‐R; pPaHC‐R with sLOC‐L, and Networks Default Mode LP‐L, Networks Default Mode PCC, Precuneous, and ICC‐L; Networks Language pSTG‐R with TP‐R, Networks Default Mode LP‐L, and Networks Default Mode LP‐R was decreased (blue lines; Figure 4, Table 2).

In post‐DBS, FC changes were correlated with UPDRS‐III improvement, including toMTG‐R ‐ aSTG L (r = 0.63; *p* = 0.0096**) and SMA‐L ‐ Networks FrontoParietal PPC ‐L (r = −0.64; *p* = 0.0109**). Motor improvement was correlated with an FC decrease between toMTG‐R and aSTG‐L, and FC increase between SMA‐L and Networks FrontoParietal PPC‐L (Figure 3C, D).

### 3.4 Diffusion tensor imaging TBSS in patients at pre‐ and post‐DBS

**FIGURE 5:**
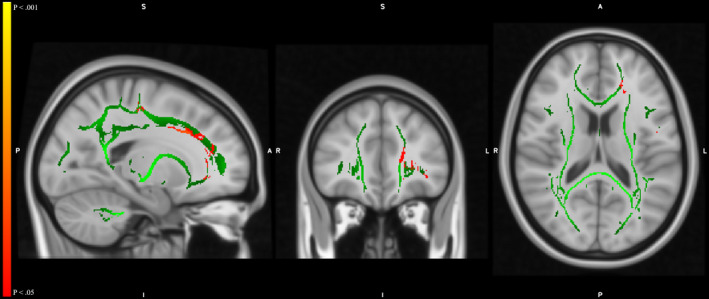
Fractional anisotropy (FA) decreased in the left anterior corona radiata at MNI coordinate x, y, and z (−19, 36, and 16) in post‐DBS patients compared with pre‐DBS (red color: pre‐DBS >post‐DBS). Green color: mean skeletal fiber skeletal after multiple comparisons by threshold‐free cluster enhancement (TFCE) *p* < 0.05

We found a significant reduction in the FA of the white matter tract in the left anterior corona radiata (ACR) located at MIN coordinate x, y, and z (−19, 36, and 16) in post‐DBS patients compared with their pre‐DBS condition (Figure 5, Table 3). The MD and radial and axial diffusivities of the patients in the post‐DBS state showed no difference compared with those in the pre‐DBS state.

**TABLE 3:** Fractional anisotropy (FA) decreased in the left anterior corona Radiata, located at specific MNI coordinates, in post‐DBS patients as compared with pre‐DBS

Contrast	WM tract	Brain region(s)	MNI (mm)	voxel size	*p*
			x, y, z		
pre‐DBS >post‐DBS (FA)	Anterior corona Radiata L	Left cerebral white matter	−19, 36, 16	692	<0.05
	Anterior corona Radiata L	Left cerebral white matter	−17, 20, 30	535	<0.05
	Anterior corona Radiata L	Left cerebral white matter	−20, 33, −7	155	<0.05

## 4 DISCUSSION

**FIGURE 6:**
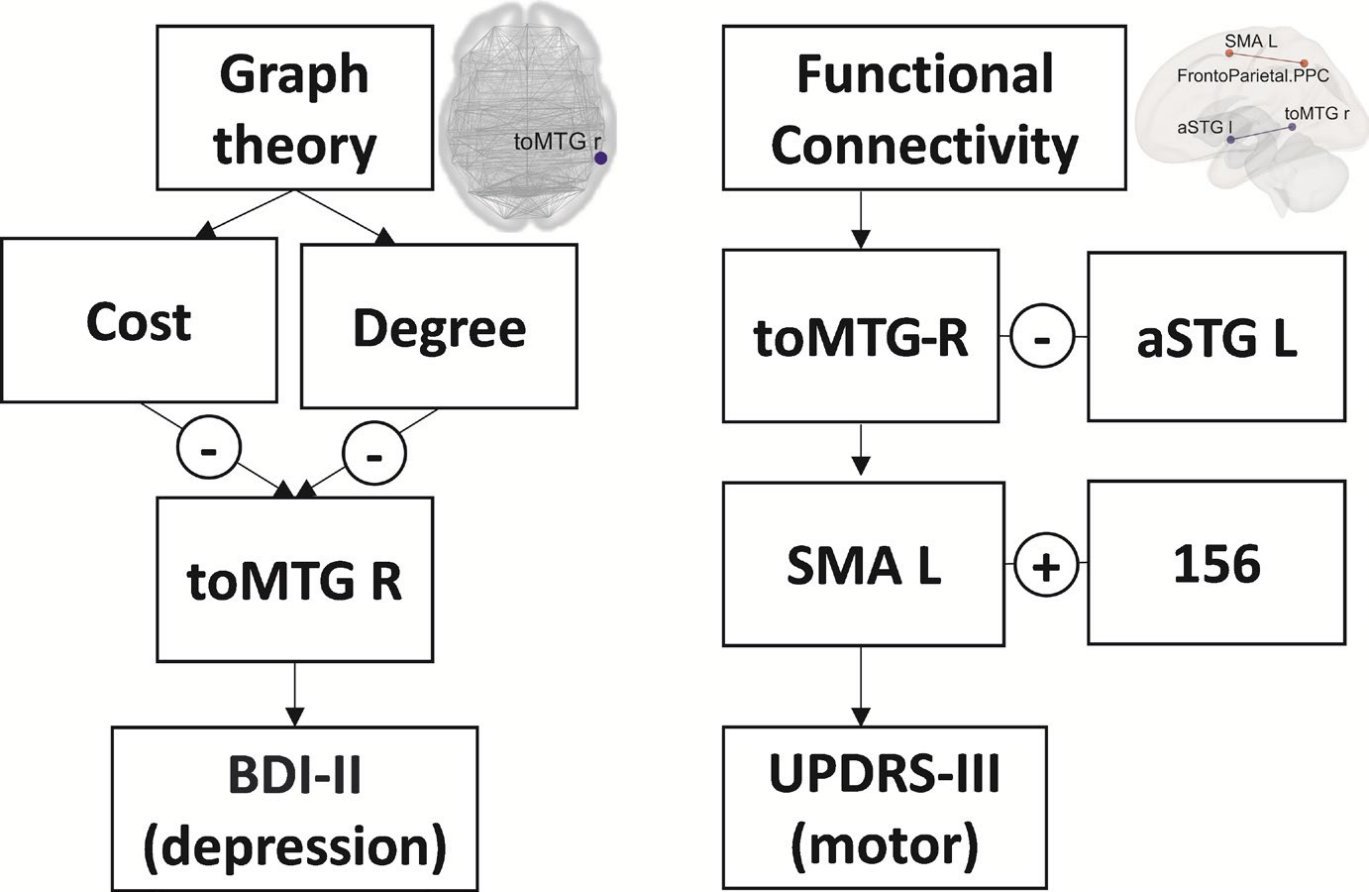
Brain topological metrics and functional connectivity changes after DBS correlated with regions associated with clinical assessment improvement. *toMTG‐R = Middle Temporal Gyrus, temporo‐occipital part‐Right; aSTG L = Superior Temporal Gyrus, anterior division Left; SMA L = Supplementary Motor Cortex‐Left; 156: networks. FrontoParietal‐PPC L. +: increase; ‐: decrease

Recent concepts of neurodegenerative disease have emphasized the importance of specific changes in brain connectivity in pathophysiology and effectiveness of treatment.^17^, ^31^, ^42^, ^43^, ^44^ In this study, we showed that “an increased connectivity of motor hubs with the brain stem and putamen” indicates that both the brain stem and putamen play a crucial role in the effects of STN‐DBS on motor improvement in PD patients.^2^, ^11^, ^20^ The FC changes in toMTG‐R‐aSTG L and SMA L‐Networks‐FrontoParietal‐PPC L are correlated with motor improvement. Furthermore, “a major decrease in the degree and cost in the toMTG‐R” indicated that connection density and centrality were decreased in the toMTG‐R and correlated with depression improvement, which could be related to the deterioration in language and speech function in PD patients after DBS.^45^ We also found a trend of cognition decline in PD patients after DBS, which could be observed in terms of topological metrics^46^ and that the decreased FA in the white matter tract was more likely to be related to the emotion and executive attention decline in these patients.^47^ In Figure 6, we schematically summarize the critical results of the study.

### 4.1 Post‐DBS motor improvement and FC

In line with the improved motor control and reduced LEDD that is widely noted in PD patients after DBS,^5^ we also found significant improvements in the UPDRS‐III motor score and a reduction in LEDD after DBS. Figure 4 shows four ROIs with increases and four with decreases in brain FC. The brainstem constitutes a central communication hub; increases in its FC with the bilateral putamen would particularly support motor improvement. FC increased in the following four regions: Language IFG, SMA‐L, SubCalc, and the brainstem. When the brainstem acts as a seed, connections increased in four directions, namely, with both sides of the putamen, the Networks Visual Lateral‐R, and the OFusG‐R (Figure 4). This indicates that the brainstem and putamen may play essential roles in the modulation of brain networks by stimulation resulting in sustained motor improvement. These findings also highlight the emphasis on the brainstem and cerebellum in PD research.^20^ In post‐DBS, FC change was correlated with UPDRS‐III improvement, including toMTG‐R‐aSTG L and SMA L‐networks‐FrontoParietal‐PPC L connections, which suggests FC change was associated with the effectiveness of STN‐DBS (Figure 3C, D).

### 4.2 Depression improvement and topological metrics: The toMTG‐R and related findings

The cost and degree of topological metrics in toMTG‐R were decreased after STN‐DBS and were correlated with an improvement in depression as interpreted by the Beck Depression Intervention (BDI‐Ⅱ) score (Figure 3A,B). Ye et al.^48^ used rs‐fMRI and graph theory for analyzing patients with major depressive disorder and found that patients had higher node centralities than normal control group. Our result showed STN‐DBS improves patients' depression and decreased these pathologically higher parameters through analyzing graph‐theory metrics. These findings indicate that the toMTG‐R may be a biomarker for depression improvement when using topological metrics as diagnostic tools. Previous studies have demonstrated a gradual decline in cognitive function after long‐term DBS.^3^, ^49^ The toMTG are related to higher‐order cognitive function in visual perception.^50^ Although our results showed a mild decline in cognitive function, the difference was not statistically significant. This may be due to the shorter follow‐ups of STN‐DBS.

### 4.3 Deterioration in language and speech function and FC

Clinically, the worsening of PD symptoms, including akinesia, speech, postural stability, freezing of gait, and cognitive function, may occur after DBS.^3^ We showed a decline in language and speech function, although this was not statistically significant, and may be attributed to the relatively short‐term follow‐up period. Nevertheless, changes in FC may reflect the deterioration in their earlier stage.

While FC decreased in post‐DBS PD patients in four brain regions, including the pMTG‐R, toMTG‐R, pPaHC‐R, and Networks, language pSTG‐R, and particularly the first three regions, may be a central hub in the decrease in FC after DBS. The MTG is related to language inputs. The pMTG is related to language^51^ and semantic memory processing.^50^ The IFG (Broca area) is related to language processing, speech production, and executive function.^50^ The left inferior frontal gyrus (IFG‐L) to the left posterior middle temporal gyrus (pMTG‐L) connection suggests top‐down influences of the frontal cortex on retrieval of semantic representations. The MTG provides relevant associations in verbal semantic memory for the IFG to perform retrieval. The left fusiform gyrus to the pMTG‐L suggests bottom‐up orthographic influences on semantic representations. Cereb7‐L, which has on posterior to primary somatosensory cortex and above the occipital lobe, is related to visual‐motor coordination and language activation. The increase in activity from the MTG and output from Cereb7‐L may cause stuttering in PD patients after DBS stimulation.^52^, ^53^, ^54^, ^55^ This finding of stuttering in PD patients may correlate with our clinical observation (UPDRS‐Ⅱ) of decreased language fluency post‐DBS.^56^

Notably, rs‐fMRI studies in PD patients showed various patterns of FC alterations, which can be attributed to the inclusion of cases at different stages of the disease and with varying durations of stimulation.

### 4.4 Emotion and executive attention and white matter fractional anisotropy: FA decrease in the ACR

The reduced FA value indicates either damage to the white matter or a loss of white matter structural integrity, which may reduce information transfer. In our findings in post‐DBS PD patients, the white matter FA values decreased in the ACR in the left hemisphere (Figure 5). The ACR is part of the limbic‐thalamo‐cortical circuitry associated with impaired top‐down emotion regulation systems and the motor pathway.^47^, ^57^, ^58^ It is also associated with executive attention network functions.^59^ Decreased FA in the ACR, therefore, might contribute to prefrontal cortex dysfunction, which is associated with inattention. Interestingly, decreased FA in the ACR occurred in the left hemisphere alone; the physiological significance thereof warrants further study. Nevertheless, damage to the ACR during the surgical procedure cannot be excluded. Currently, the non‐motor PD symptoms of emotional and executive attention deficits are receiving increasing attention. Many behavioral assessments have been developed and used to evaluate emotion and executive attention.^60^ Using the rs‐fMRI and behavioral assessments to explore the effects of DBS on emotion and executive attention is, therefore, both informative and valuable.

There are several limitations to this study. First, a major issue in fMRI with DBS is the occurrence of geometric distortions and drop‐out of the EPI signal in the vicinity of the electrodes, particularly near the skull where electrodes are connected to the extension leads; these problems are currently unavoidable.^17^ Furthermore, the impact of DBS‐induced artifacts on the rs‐fMRI signal has not been investigated in detail, and more methodological work is required to address potential issues. Second, according to our objective, we needed to turn DBS off 10 min before the scan due to safety concerns.^61^ We defined this post‐DBS_ON/MedON_ state as the highest “on” state, in which DBS was switched on and the patient was on medications. Moreover, our included patients remained in their “on” state during the scan. This sustained effect observed after DBS was turned off could be due to synaptic plasticity within the stimulated neural network.^45^ Third, the results could also be confounded by pharmacodynamic changes produced by the significant reduction in medication. Lastly, patients had different stimulation parameters, which could cause diversity of results. Furthermore, the length of the follow‐up times after DBS was not uniform, making it difficult to compare the result with other studies.

In conclusion, we demonstrated that STN‐DBS alters graph‐theoretical metrics, FC, and white matter integrity, leading to a significant improvement in the motor and psychiatric symptoms of patients with PD. The changes in brain connectivity from this multi‐modal imaging were also associated with the extent of postoperative clinical improvement. These results suggest that changes in brain networks might explain the benefit of STN‐DBS in PD.

## CONFLICT OF INTEREST

All authors declare that they have no competing interests.

## AUTHOR CONTRIBUTIONS

Li‐Chuan Huang performed the experiments, analyzed the rs‐fMRI data, and wrote the manuscript. Li‐Guo Chen analyzed the DTI data. Ping‐An Wu, Cheng‐Yoong Pang, and Shinn‐Zong Lin contributed conception and design of the study. Sheng‐Tzung Tsai was responsible for the study design, data review, editing, and revision of the manuscript. Shin‐Yuan Chen reviewed and revised the manuscript and was responsible for the supervision of the study concept and design, critical revision of the manuscript, and study supervision. All authors contributed to manuscript revision and read and approved the submitted version.

## Supporting information

Table S1

Table S2

## Data Availability

The data that support the findings of this study are available from the corresponding author upon reasonable request.
