# Supplementary material for: Effect of deep brain stimulation on brain network and white matter integrity in Parkinson's disease
Source: CNS Neurosci Ther. 2021 Oct 12;28(1):92–104. doi: 10.1111/cns.13741 (PMC8673709; doi:10.1111/cns.13741)
Supplement: Supplementary file 1 — Table S1 [file CNS-28-92-s002.docx]

Supplementary Table s1. DBS STN target coordinates.

|  |  | Pre-surgery | | | | | |  | MER | | | | | | |  | |
| --- | --- | --- | --- | --- | --- | --- | --- | --- | --- | --- | --- | --- | --- | --- | --- | --- | --- |
|  |  | Right STN | | | Left STN | | |  | Right STN | | | | Left STN | | |  | |
| Patient | AC-PC | X | Y | Z | X | Y | Z | AC-PC | X | Y | Z | STN depth | X | Y | Z | STN depth |  |
| 1 | 22.92 | 11.05 | -1.43 | -4.94 | -11.00 | -1.51 | -5.03 | 22.92 | 11.05 | -1.43 | -4.64 | 6.06 | -11.00 | -1.51 | -5.03 | 6.52 |  |
| 2 | 25.13 | 10.93 | -1.68 | -4.37 | -10.77 | -2.13 | -4.96 | 25.13 | 10.93 | -1.68 | -5.67 | 5.31 | -10.76 | -2.13 | -4.96 | 6.45 |  |
| 3 | 23.95 | 11.94 | -1.57 | -3.80 | -10.97 | -2.12 | -5.98 | 23.95 | 11.94 | -1.57 | -4.40 | 5.02 | -10.97 | -2.12 | -7.47 | 4.81 |  |
| 4 | 23.08 | 10.89 | -2.16 | -5.21 | -10.64 | -1.76 | -5.28 | 23.08 | 10.89 | -2.16 | -4.89 | 5.23 | -10.64 | -1.76 | -6.37 | 5.18 |  |
| 5 | 22.63 | 11.05 | -2.99 | -5.00 | -10.95 | -2.49 | -5.73 | 22.63 | 11.05 | -2.99 | -6.25 | 5.78 | -10.95 | -2.49 | -6.04 | 6.08 |  |
| 6 | 21.50 | 10.27 | -4.00 | -5.22 | -10.22 | -4.23 | -4.39 | 21.50 | 10.27 | -4.00 | -5.15 | 5.97 | -10.22 | -4.23 | -4.71 | 7.03 |  |
| 7 | 24.31 | 11.27 | -1.78 | -5.70 | -11.23 | -2.18 | -5.71 | 24.31 | 11.27 | -1.78 | -6.70 | 5.93 | -11.23 | -2.18 | -5.87 | 5.77 |  |
| 8 | 24.76 | 10.76 | -2.54 | -4.88 | -10.37 | -1.31 | -5.17 | 24.76 | 10.76 | -2.54 | -6.11 | 6.70 | -10.37 | -1.31 | -6.47 | 5.52 |  |
| 9 | 22.07 | 10.63 | -2.41 | -5.09 | -10.40 | -1.79 | -5.55 | 22.07 | 10.63 | -2.41 | -5.09 | 5.84 | -10.40 | -1.79 | -5.51 | 5.66 |  |
| 10 | 21.35 | 10.58 | -3.43 | -4.65 | -10.42 | -2.47 | -4.23 | 21.35 | 10.58 | -3.43 | -4.65 | NA | -10.42 | -2.47 | -4.39 | 5.16 |  |
| 11 | 23.53 | 11.77 | -2.67 | -5.89 | -11.24 | -2.59 | -6.18 | 23.53 | 11.77 | -2.67 | -6.66 | 5.14 | -11.24 | -2.59 | -6.58 | 5.65 |  |
| 12 | 22.11 | 11.68 | -1.44 | -5.30 | -12.32 | -1.43 | -4.94 | 22.11 | 11.68 | -1.44 | -5.16 | 6.07 | -12.32 | -1.43 | -5.44 | 5.97 |  |
| 13 | 23.16 | 11.02 | -2.33 | -5.95 | -11.02 | -2.91 | -5.86 | 23.16 | 11.02 | -2.33 | -5.95 | NA | -11.02 | -2.91 | -5.43 | 5.18 |  |
| 14 | 23.66 | 11.69 | -2.66 | -5.32 | -11.80 | -3.21 | -4.81 | 23.66 | 11.69 | -2.66 | -6.64 | 5.50 | -11.80 | -3.21 | -5.71 | 5.44 |  |
| 15 | 22.40 | 12.68 | -1.71 | -5.24 | -11.3 | -1.71 | -4.96 | 22.40 | 12.68 | -1.71 | -4.54 | 6.65 | -11.34 | -1.71 | -4.02 | 6.20 |  |

Abbreviations: STN: subthalamic nucleus; AC-PC: anterior commissure - posterior commissure;

MER: microelectrode recording; NA: not available.
