# Supplementary material for: Effect of deep brain stimulation on brain network and white matter integrity in Parkinson's disease
Source: CNS Neurosci Ther. 2021 Oct 12;28(1):92–104. doi: 10.1111/cns.13741 (PMC8673709; doi:10.1111/cns.13741)
Supplement: Supplementary file 2 — Table S2 [file CNS-28-92-s001.docx]

**Supplementary table 2: Conn toolbox defined atlas and networks ROIs of brain.**

| ROIs | Atlas/Networks | Abbreviation | Coordinate |
| --- | --- | --- | --- |
| 1 | atlas.FP r | Frontal Pole Right |  |
| 2 | atlas.FP l | Frontal Pole Left |  |
| 3 | atlas.IC r | Insular Cortex Right |  |
| 4 | atlas.IC l | Insular Cortex Left |  |
| 5 | atlas.SFG r | Superior Frontal Gyrus Right |  |
| 6 | atlas.SFG l | Superior Frontal Gyrus Left |  |
| 7 | atlas.MidFG r | Middle Frontal Gyrus Right |  |
| 8 | atlas.MidFG l | Middle Frontal Gyrus Left |  |
| 9 | atlas.IFG tri r | Inferior Frontal Gyrus, pars triangularis Right |  |
| 10 | atlas.IFG tri l | Inferior Frontal Gyrus, pars triangularis Left |  |
| 11 | atlas.IFG oper r | Inferior Frontal Gyrus, pars opercularis Right |  |
| 12 | atlas.IFG oper l | Inferior Frontal Gyrus, pars opercularis Left |  |
| 13 | atlas.PreCG r | Precentral Gyrus Right |  |
| 14 | atlas.PreCG l | Precentral Gyrus Left |  |
| 15 | atlas.TP r | Temporal Pole Right |  |
| 16 | atlas.TP l | Temporal Pole Left |  |
| 17 | atlas.aSTG r | Superior Temporal Gyrus, anterior division Right |  |
| 18 | atlas.aSTG l | Superior Temporal Gyrus, anterior division Left |  |
| 19 | atlas.pSTG r | Superior Temporal Gyrus, posterior division Right |  |
| 20 | atlas.pSTG l | Superior Temporal Gyrus, posterior division Left |  |
| 21 | atlas.aMTG r | Middle Temporal Gyrus, anterior division Right |  |
| 22 | atlas.aMTG l | Middle Temporal Gyrus, anterior division Left |  |
| 23 | atlas.pMTG r | Middle Temporal Gyrus, posterior division Right |  |
| 24 | atlas.pMTG l | Middle Temporal Gyrus, posterior division Left |  |
| 25 | atlas.toMTG r | Middle Temporal Gyrus, temporooccipital part Right |  |
| 26 | atlas.toMTG l | Middle Temporal Gyrus, temporooccipital part Left |  |
| 27 | atlas.aITG r | Inferior Temporal Gyrus, anterior division Right |  |
| 28 | atlas.aITG l | Inferior Temporal Gyrus, anterior division Left |  |
| 29 | atlas.pITG r | Inferior Temporal Gyrus, posterior division Right |  |
| 30 | atlas.pITG l | Inferior Temporal Gyrus, posterior division Left |  |
| 31 | atlas.toITG r | Inferior Temporal Gyrus, temporooccipital part Right |  |
| 32 | atlas.toITG l | Inferior Temporal Gyrus, temporooccipital part Left |  |
| 33 | atlas.PostCG r | Postcentral Gyrus Right |  |
| 34 | atlas.PostCG l | Postcentral Gyrus Left |  |
| 35 | atlas.SPL r | Superior Parietal Lobule Right |  |
| 36 | atlas.SPL l | Superior Parietal Lobule Left |  |
| 37 | atlas.aSMG r | Supramarginal Gyrus, anterior division Right |  |
| 38 | atlas.aSMG l | Supramarginal Gyrus, anterior division Left |  |
| 39 | atlas.pSMG r | Supramarginal Gyrus, posterior division Right |  |
| 40 | atlas.pSMG l | Supramarginal Gyrus, posterior division Left |  |
| 41 | atlas.AG r | Angular Gyrus Right |  |
| 42 | atlas.AG l | Angular Gyrus Left |  |
| 43 | atlas.sLOC r | Lateral Occipital Cortex, superior division Right |  |
| 44 | atlas.sLOC l | Lateral Occipital Cortex, superior division Left |  |
| 45 | atlas.iLOC r | Lateral Occipital Cortex, inferior division Right |  |
| 46 | atlas.iLOC l | Lateral Occipital Cortex, inferior division Left |  |
| 47 | atlas.ICC r | Intracalcarine Cortex Right |  |
| 48 | atlas.ICC l | Intracalcarine Cortex Left |  |
| 49 | atlas.MedFC | Frontal Medial Cortex |  |
| 50 | atlas.SMA r | Juxtapositional Lobule Cortex -formerly Supplementary Motor Cortex- Right |  |
| 51 | atlas.SMA l | Juxtapositional Lobule Cortex -formerly Supplementary Motor Cortex- Left |  |
| 52 | atlas.SubCalC | Subcallosal Cortex |  |
| 53 | atlas.PaCiG r | Paracingulate Gyrus Right |  |
| 54 | atlas.PaCiG l | Paracingulate Gyrus Left |  |
| 55 | atlas.AC | Cingulate Gyrus, anterior division |  |
| 56 | atlas.PC | Cingulate Gyrus, posterior division |  |
| 57 | atlas.Precuneous | Precuneous Cortex |  |
| 58 | atlas.Cuneal r | Cuneal Cortex Right |  |
| 59 | atlas.Cuneal l | Cuneal Cortex Left |  |
| 60 | atlas.FOrb r | Frontal Orbital Cortex Right |  |
| 61 | atlas.FOrb l | Frontal Orbital Cortex Left |  |
| 62 | atlas.aPaHC r | Parahippocampal Gyrus, anterior division Right |  |
| 63 | atlas.aPaHC l | Parahippocampal Gyrus, anterior division Left |  |
| 64 | atlas.pPaHC r | Parahippocampal Gyrus, posterior division Right |  |
| 65 | atlas.pPaHC l | Parahippocampal Gyrus, posterior division Left |  |
| 66 | atlas.LG r | Lingual Gyrus Right |  |
| 67 | atlas.LG l | Lingual Gyrus Left |  |
| 68 | atlas.aTFusC r | Temporal Fusiform Cortex, anterior division Right |  |
| 69 | atlas.aTFusC l | Temporal Fusiform Cortex, anterior division Left |  |
| 70 | atlas.pTFusC r | Temporal Fusiform Cortex, posterior division Right |  |
| 71 | atlas.pTFusC l | Temporal Fusiform Cortex, posterior division Left |  |
| 72 | atlas.TOFusC r | Temporal Occipital Fusiform Cortex Right |  |
| 73 | atlas.TOFusC l | Temporal Occipital Fusiform Cortex Left |  |
| 74 | atlas.OFusG r | Occipital Fusiform Gyrus Right |  |
| 75 | atlas.OFusG l | Occipital Fusiform Gyrus Left |  |
| 76 | atlas.FO r | Frontal Operculum Cortex Right |  |
| 77 | atlas.FO l | Frontal Operculum Cortex Left |  |
| 78 | atlas.CO r | Central Opercular Cortex Right |  |
| 79 | atlas.CO l | Central Opercular Cortex Left |  |
| 80 | atlas.PO r | Parietal Operculum Cortex Right |  |
| 81 | atlas.PO l | Parietal Operculum Cortex Left |  |
| 82 | atlas.PP r | Planum Polare Right |  |
| 83 | atlas.PP l | Planum Polare Left |  |
| 84 | atlas.HG r | Heschls Gyrus Right |  |
| 85 | atlas.HG l | Heschls Gyrus Left |  |
| 86 | atlas.PT r | Planum Temporale Right |  |
| 87 | atlas.PT l | Planum Temporale Left |  |
| 88 | atlas.SCC r | Supracalcarine Cortex Right |  |
| 89 | atlas.SCC l | Supracalcarine Cortex Left |  |
| 90 | atlas.OP r | Occipital Pole Right |  |
| 91 | atlas.OP l | Occipital Pole Left |  |
| 92 | atlas.Thalamus r |  |  |
| 93 | atlas.Thalamus l |  |  |
| 94 | atlas.Caudate r |  |  |
| 95 | atlas.Caudate l |  |  |
| 96 | atlas.Putamen r |  |  |
| 97 | atlas.Putamen l |  |  |
| 98 | atlas.Pallidum r |  |  |
| 99 | atlas.Pallidum l |  |  |
| 100 | atlas.Hippocampus r |  |  |
| 101 | atlas.Hippocampus l |  |  |
| 102 | atlas.Amygdala r |  |  |
| 103 | atlas.Amygdala l |  |  |
| 104 | atlas.Accumbens r |  |  |
| 105 | atlas.Accumbens l |  |  |
| 106 | atlas.Brain-Stem |  |  |
| 107 | atlas.Cereb1 l | Cerebelum Crus1 Left |  |
| 108 | atlas.Cereb1 r | Cerebelum Crus1 Right |  |
| 109 | atlas.Cereb2 l | Cerebelum Crus2 Left |  |
| 110 | atlas.Cereb2 r | Cerebelum Crus2 Right |  |
| 111 | atlas.Cereb3 l | Cerebelum 3 Left |  |
| 112 | atlas.Cereb3 r | Cerebelum 3 Right |  |
| 113 | atlas.Cereb45 l | Cerebelum 4 5 Left |  |
| 114 | atlas.Cereb45 r | Cerebelum 4 5 Right |  |
| 115 | atlas.Cereb6 l | Cerebelum 6 Left |  |
| 116 | atlas.Cereb6 r | Cerebelum 6 Right |  |
| 117 | atlas.Cereb7 l | Cerebelum 7b Left |  |
| 118 | atlas.Cereb7 r | Cerebelum 7b Right |  |
| 119 | atlas.Cereb8 l | Cerebelum 8 Left |  |
| 120 | atlas.Cereb8 r | Cerebelum 8 Right |  |
| 121 | atlas.Cereb9 l | Cerebelum 9 Left |  |
| 122 | atlas.Cereb9 r | Cerebelum 9 Right |  |
| 123 | atlas.Cereb10 l | Cerebelum 10 Left |  |
| 124 | atlas.Cereb10 r | Cerebelum 10 Right |  |
| 125 | atlas.Ver12 | Vermis 1 2 |  |
| 126 | atlas.Ver3 | Vermis 3 |  |
| 127 | atlas.Ver45 | Vermis 4 5 |  |
| 128 | atlas.Ver6 | Vermis 6 |  |
| 129 | atlas.Ver7 | Vermis 7 |  |
| 130 | atlas.Ver8 | Vermis 8 |  |
| 131 | atlas.Ver9 | Vermis 9 |  |
| 132 | atlas.Ver10 | Vermis 10 |  |
| 133 | networks.DefaultMode.MPFC |  | 1, 55, -3 |
| 134 | networks.DefaultMode.LP | L | -39, -77, 33 |
| 135 | networks.DefaultMode.LP | R | 47, -67, 29 |
| 136 | networks.DefaultMode.PCC |  | 1, -61, 38 |
| 137 | networks.SensoriMotor.Lateral | L | -55, -12, 29 |
| 138 | networks.SensoriMotor.Lateral | R | 56, -10, 29 |
| 139 | networks.SensoriMotor.Superior |  | 0, -31, 67 |
| 140 | networks.Visual.Medial |  | 2, -79, 12 |
| 141 | networks.Visual.Occipital |  | 0, -93, -4 |
| 142 | networks.Visual.Lateral | L | -37, -79, 10 |
| 143 | networks.Visual.Lateral | R | 38, -72, 13 |
| 144 | networks.Salience.ACC |  | 0, 22, 35 |
| 145 | networks.Salience.AInsula | L | -44, 13, 1 |
| 146 | networks.Salience.AInsula | R | 47, 14, 0 |
| 147 | networks.Salience.RPFC | L | -32, 45, 27 |
| 148 | networks.Salience.RPFC | R | 32, 46, 27 |
| 149 | networks.Salience.SMG | L | -60, -39, 31 |
| 150 | networks.Salience.SMG | R | 62, -35, 32 |
| 151 | networks.DorsalAttention.FEF | L | -27, -9, 64 |
| 152 | networks.DorsalAttention.FEF | R | 30, -6, 64 |
| 153 | networks.DorsalAttention.IPS | L | -39, -43, 52 |
| 154 | networks.DorsalAttention.IPS | R | 39, -42, 54 |
| 155 | networks.FrontoParietal.LPFC | L | -43, 33, 28 |
| 156 | networks.FrontoParietal.PPC | L | -46, -58, 49 |
| 157 | networks.FrontoParietal.LPFC | R | 41, 38, 30 |
| 158 | networks.FrontoParietal.PPC | R | 52, -52, 45 |
| 159 | networks.Language.IFG | L | -51, 26, 2 |
| 160 | networks.Language.IFG | R | 54, 28, 1 |
| 161 | networks.Language.pSTG | L | -57, -47, 15 |
| 162 | networks.Language.pSTG | R | 59, -42, 13 |
| 163 | networks.Cerebellar.Anterior |  | 0, -63, -30 |
| 164 | networks.Cerebellar.Posterior |  | 0, -79, -32 |
| 165 | Grey Matter |  |  |

- **ROIS defined from:**

1. Cortical ROIs from FSL Harvard-Oxford Atlas maximum likelihood cortical atlas (HarvardOxford-cort-maxprob-thr25-1mm.nii); divided bilateral areas into left/right hemisphere; (91 ROIs)
2. Subcortical ROIs from FSL Harvard-Oxford Atlas maximum likelihood subcortical atlas (HarvardOxford-sub-maxprob-thr25-1mm.nii); disregarded Cerebral White Matter, Cerebral Cortex, and Lateral Ventrical areas; (15 ROIs)
3. Cerebellar parcelation from AAL Atlas (26 ROIs)

- **Atlas of commonly used networks:**

1. Default Mode Network (4 ROIs)
2. SensoriMotor (3 ROIs)
3. Visual (4 ROIs)
4. Salience / Cingulo-Opercular (7 ROIs)
5. DorsalAttention (4 ROIs)
6. FrontoParietal / Central Executive (4 ROIs)
7. Language (4 ROIs)
8. Cerebellar (2 ROIs)

ROIs defined from CONN's ICA analyses of HCP dataset (497 subjects)
